# Improving sensitivity of linear regression-based cell type-specific differential expression deconvolution with per-gene vs. global significance threshold

**DOI:** 10.1186/s12859-016-1226-z

**Published:** 2016-10-06

**Authors:** Edmund R. Glass, Mikhail G. Dozmorov

**Affiliations:** Department of Biostatistics, Virginia Commonwealth University, School of Medicine, PO Box 980032, Richmond, VA 23298 USA

**Keywords:** Deconvolution, Linear regression, Differential expression, Cell type-specific, Sensitivity analysis

## Abstract

**Background:**

The goal of many human disease-oriented studies is to detect molecular mechanisms different between healthy controls and patients. Yet, commonly used gene expression measurements from blood samples suffer from variability of cell composition. This variability hinders the detection of differentially expressed genes and is often ignored. Combined with cell counts, heterogeneous gene expression may provide deeper insights into the gene expression differences on the cell type-specific level.

Published computational methods use linear regression to estimate cell type-specific differential expression, and a global cutoff to judge significance, such as False Discovery Rate (FDR). Yet, they do not consider many artifacts hidden in high-dimensional gene expression data that may negatively affect linear regression. In this paper we quantify the parameter space affecting the performance of linear regression (sensitivity of cell type-specific differential expression detection) on a per-gene basis.

**Results:**

We evaluated the effect of sample sizes, cell type-specific proportion variability, and mean squared error on sensitivity of cell type-specific differential expression detection using linear regression. Each parameter affected variability of cell type-specific expression estimates and, subsequently, the sensitivity of differential expression detection. We provide the R package, LRCDE, which performs linear regression-based cell type-specific differential expression (deconvolution) detection on a gene-by-gene basis. Accounting for variability around cell type-specific gene expression estimates, it computes per-gene t-statistics of differential detection, *p*-values, t-statistic-based sensitivity, group-specific mean squared error, and several gene-specific diagnostic metrics.

**Conclusions:**

The sensitivity of linear regression-based cell type-specific differential expression detection differed for each gene as a function of mean squared error, per group sample sizes, and variability of the proportions of target cell (cell type being analyzed). We demonstrate that LRCDE, which uses Welch’s *t*-test to compare per-gene cell type-specific gene expression estimates, is more sensitive in detecting cell type-specific differential expression at α < 0.05 missed by the global false discovery rate threshold FDR < 0.3.

**Electronic supplementary material:**

The online version of this article (doi:10.1186/s12859-016-1226-z) contains supplementary material, which is available to authorized users.

## Background

Detection of differential gene expression at the cell type-specific level (deconvolution) aims to provide deeper insight into underlying biological causes of a given pathology. Investigators studying disease mechanisms benefit from knowing which genes in which cell types are differentially expressed. Yet, deconvolution is complicated by the prohibitive cost of extraction of pure cell type specimens, and non-linearity of amplified pure samples [1]. Statistical methods of quantifying cell type-specific differential gene expression (CDE) are a viable alternative to deconvolve heterogeneous gene expression signal into the cell type-specific measures that can be compared for significant differences [2–4].

There are two rationales behind CDE. One is that group-wise differential expression analysis on heterogeneous measures provides no information about which cell types are the source of any detected differences [2]. The other is that differential expression detection analysis applied only to heterogeneous tissue may miss the true cell type-specific expression differences. Thus, CDE analysis may uncover cell type-specific signal not seen at the heterogeneous level [5] (Additional file 1: section 1.1).

Previous efforts primarily focused on quantifying cell proportions from heterogeneous tissue is by using *a priori* known cell signatures as predictors in a linear regression model [3, 4, 6–10]. The other, less developed approach focuses on cell specific gene expression detection. It relies on linear regression to deconvolve heterogeneous gene expression measures using cell proportions as predictors. The coefficient estimates in this setup represent average cell type-specific expression levels, comparable if two groups are analyzed [5, 10–13]. Both approaches require two pieces of information: 1) the heterogeneous gene expression measures, and 2) either the cell signatures (first approach), or cell proportions (second approach).

Two algorithms addressing the second approach have been published (csSAM, DSection) [5, 11]. The csSAM approach uses heterogeneous observations as outcomes in a linear regression model, and the measured cell proportions as predictors. Two regressions, one per study group (e.g., case–control groups), are performed and the difference between coefficient estimates represents the cell type-specific differential expression estimates. Group label permutations are performed and false discovery rates (FDR) are estimated across the range of effect sizes per cell type. The csSAM authors acknowledge that increasing sample variability will improve cell type-specific expression accuracy, and we quantified the effect of such variability.

DSection assumes that cell proportion measures are imprecise and that this imprecision must be accounted for. DSection uses a Bayesian approach to “de-noise” cell proportion measures prior to linear regression deconvolution. The authors of DSection contrast their method to a “gold standard” of using linear regression when cell proportions are precisely known. The DSection authors correctly point out that, in real settings, no exact knowledge of cell proportions is known and that measurements are presumed to be estimates. They also acknowledge that the choice of prior information to use with their Bayesian approach has a subjective component.

**Fig. 1:**
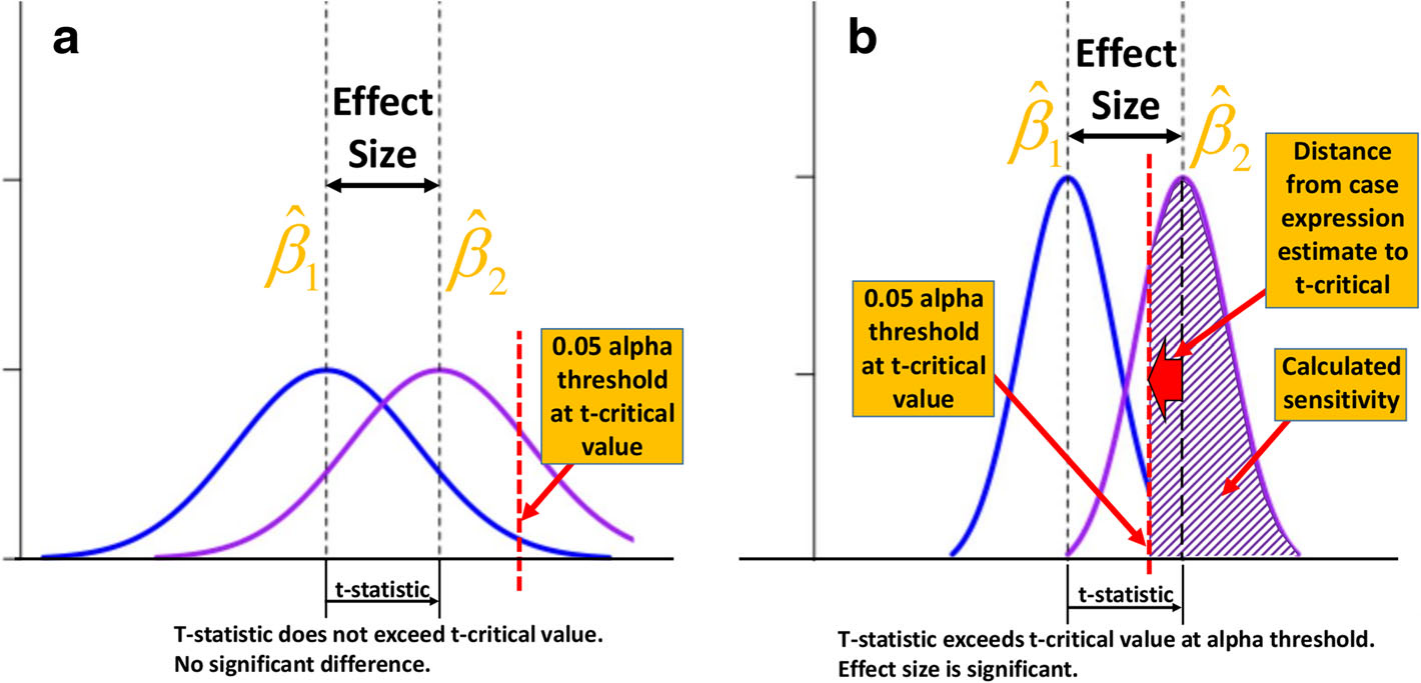
Differential expression detection sensitivity is primarily affected by two factors: cell type-specific expression estimate (point estimate) variability and cell type-specific differential expression (**a**). A two-sample t-statistic is computed using the observed effect size (cell type-specific differential expression (Additional file 1: section 1.3). If the t-statistic does not exceed the t-critical value, which is based on the alpha significance threshold, then we cannot conclude that a significant difference has been observed between the two groups. Given an observed difference which is determined to be significant, then we may reject the null hypothesis of no difference between controls and cases and calculate sensitivity for this observed difference, based upon the distance from the case group expression estimate to the t-critical value (**b**). Bell curves represent distribution of cell type-specific expression estimates (point estimates - vertical dashed lines). The cell type-specific differential expression estimate (effect size) corresponds to the distance between vertical dashed lines for cases and controls (blue/purple bell curves, respectively)

**Fig. 2:**
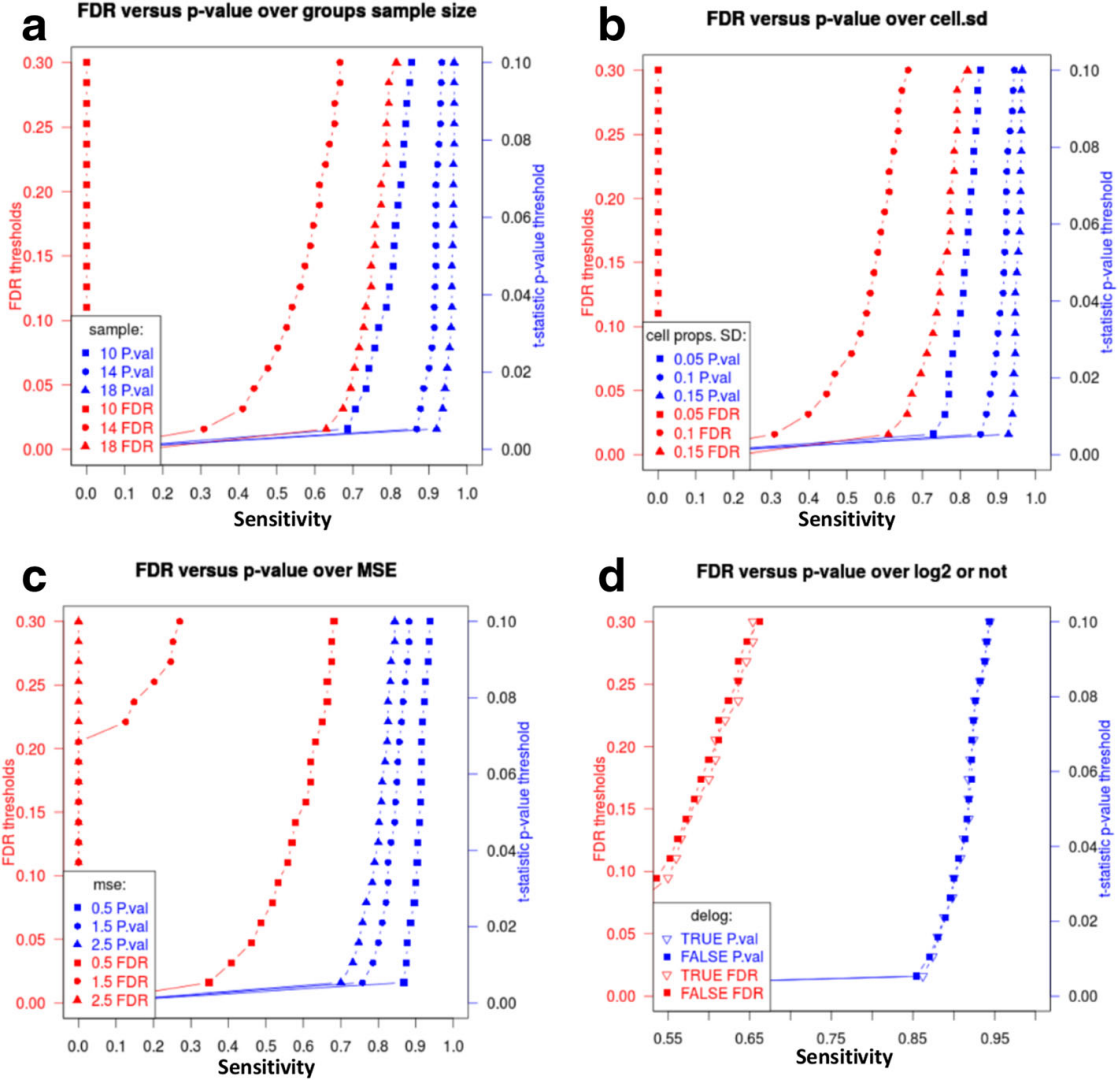
Parameters affecting sensitivity of cell type-specific differential expression detection. The effect of (**a**) per-group samples sizes (10, 14, and 18); (**b**) cell proportion SD (0.05, 0.1, and 0.15); (**c**) MSE (0.5, 1.5, and 2.5); (**d**) log2-transformation vs. as-is data. Unless specified otherwise, log2-transformed data was used, and the following parameters were held constant: per-group sample size - 14, condition number - 100, cell proportion SD - 0.1 (0.6 for C), MSE - 1.5

In this study, we investigated the sensitivity of linear regression to detect cell type-specific gene expression differences on per-gene basis. Parameters affecting the variability of cell type-specific expression estimates (Fig. 1), and the sensitivity of cell type-specific differential expression, include, 1) sample size per study group, 2) average spread of heterogeneous measures around a linear regression prediction fit (size of residuals, quantified by mean squared error – MSE), and 3) variability of cell type-specific proportions across samples. We tested the effect of each parameter in simulation settings, while controlling other parameters (Fig. 2). For fixed values of sample size and cell proportion variability, any modification of MSE or cell type-specific differential expression affected the sensitivity of LRCDE. Since MSE and cell type-specific differential expression are gene-dependent, we conclude that any evaluation of sensitivity of cell type-specific differential expression detection must be assessed on per-gene basis, instead of a global significance threshold. We implement our approach in the LRCDE R package that utilizes variability of the per-gene cell type-specific expression estimates, and is more sensitive in detecting true cell type-specific differentially expressed genes as compared with the global significance cutoff.

## Methods

### Modeling heterogeneous gene expression measures using linear regression (LR) given sample specific cell proportions (deconvolution)

We model heterogeneous gene expression measures across samples as cumulative contributions of cell type-specific gene expression measures weighted by the corresponding cell proportions of *P* cell types. A biologically meaningful constraint of this model is that cell proportions for any given sample should sum up to 1, or 100 % [5]. As proposed, heterogeneous gene expression measures (*y*_*mn*_, where *n* is gene index, *m* is sample index) are modeled using a linear regression approach:

1 $$ {y}_{mn}={\displaystyle {\sum}_{k=1}^p{\beta}_{kn}{x}_{km}+{\varepsilon}_{mn}} $$

where *β*_*kn*_ is the average theoretical cell type-specific gene expression for the *k* ^*th*^ of *p* total cell types, *x*_*km*_ is the cell proportion (predictor), and *ε*_*mn*_ is a normally distributed random error defined as the difference between the observed values *y*_*mn*_ and values predicted by the linear regression *ŷ*_*mn*_, (*y*_*mn*_ − *ŷ*_*mn*_). This allows obtaining linear regression coefficient estimates ${\widehat{\beta}}_{kn}$, interpreted as cell type-specific gene expression estimates. Intuitively, eq. 1 describes a linear relationship between heterogeneous gene expression level *y*_*mn*_ and contribution of cell type-specific gene expression estimates ${\widehat{\beta}}_{kn}$ weighted by the corresponding cell proportions *x*_*km*_. The model in eq. 1 contains no intercept term since we assume zero heterogeneous expression (*y*_*mn*_ *=* 0) in the absence of individual cell contributions. Thus, for each gene we have a total of *P* cell type-specific gene expression estimates (regression coefficients, one per cell type) in the model. Model in eq. 1 is more compactly represented in matrix form, for a single gene *j*:

2 $$ {\mathbf{y}}_j=\mathbf{X}{\boldsymbol{\upbeta}}_j+\boldsymbol{\upvarepsilon} $$

The matrix form eq. 2 suggests the form in which **β**_*j*_ is estimated:

3 $$ {\widehat{\boldsymbol{\upbeta}}}_j={\left(\mathbf{X}\mathbf{\hbox{'}}\mathbf{X}\right)}^{-1}\mathbf{X}\mathbf{\hbox{'}}{\mathbf{y}}_j $$

Fitted regression estimates are then given by:

4 $$ {\widehat{\mathbf{y}}}_j=\mathbf{X}{\widehat{\boldsymbol{\upbeta}}}_j $$

which are required in order to calculate residual values needed to estimate the variance of ${\widehat{\boldsymbol{\upbeta}}}_j$.

Obtaining cell type-specific gene expression estimates carries a quantifiable level of uncertainty. This uncertainty can be expressed as a function of sample size, number of cell types, the size of the residuals, and the variability of cell type proportions. The formula for the theoretical variance of the linear regression coefficient for simple linear regression (single predictor vector ***X***) provides an intuitive illustration of how various parameters affect the variance:

5 $$ \operatorname{var}\left({\widehat{\beta}}_1\right)=\frac{\sigma^2}{{\displaystyle {\sum}_{i=1}^m{\left({x}_i-\overline{x}\right)}^2}} $$

In practice, the estimated variance of ${\widehat{\beta}}_1$ in eq. 5 uses the mean squared error (MSE) as an estimate of *σ* ^2^, represented as *s* ^2^:

6 $$ {s}^2=MSE=\frac{{\displaystyle {\sum}_{i=1}^m{\left({y}_i-{\widehat{y}}_i\right)}^2}}{\left(M-P\right)} $$

In this simple linear regression context of eq. 5 and eq. 6, *M* is the sample size and *P* is typically equal to 2, since there are two parameters being estimated: an intercept term ${\widehat{\beta}}_0$ and the coefficient of the predictor variable: ${\widehat{\beta}}_1$. Thus, the estimated variance of ${\widehat{\beta}}_1$ in simple linear regression is represented as:

7 $$ \widehat{\operatorname{var}{\widehat{\beta}}_1}=\frac{{\displaystyle {\sum}_{i=1}^m{\left({y}_i-{\widehat{y}}_i\right)}^2}/\left(M-P\right)}{{\displaystyle {\sum}_{i=1}^m{\left({x}_i-\overline{x}\right)}^2}} $$

where *y*_*i*_ − *ŷ*_*i*_ is the residual for sample *i*, and ${x}_i-\overline{x}$ is the difference between the predictor for sample *i* and the mean of *x* across all *M* samples. In this way, predictor variability is captured in the denominator of eq. 7, as is sample size M. Residual variability is captured in the numerator of eq. 7. Each component of eq. 7 affects the estimated variance of ${\widehat{\beta}}_1$ (Additional file 1: section 1.2).

In multivariate linear regression, matrix notation simplifies representation of the variances of all *P* regression coefficients. The theoretical variance-covariance matrix Σ of linear regression coefficients is represented as:

8 $$ \sum ={\sigma}^2\left(\mathbf{X}\mathbf{\hbox{'}}{\mathbf{X}}^{-1}\right) $$

where the variances of the *k* ^*th*^ individual ${\widehat{\beta}}_k$ regression coefficients are found on the diagonal of Σ. In eq. 8 it is less intuitive to see the way in which individual parameters affect the variances of the individual ${\widehat{\beta}}_k$ in matrix form, yet the principles are the same as in eq. 5. Predictor variability is captured in the inverse of the design matrix: (**X** ' **X**)^− 1^, analogous to the denominator of eq. 5.

As with simple single variable regression, *σ* ^2^ is estimated by MSE, represented by *s* ^2^ providing the estimated covariance matrix:

9 $$ \widehat{\sum}={s}^2\left(\mathbf{X}\mathbf{\hbox{'}}{\mathbf{X}}^{-1}\right) $$

where *s* ^2^ is:

10 $$ {s}^2=\frac{\left(\mathbf{y}-\mathbf{X}\widehat{\boldsymbol{\upbeta}}\right)\mathbf{\hbox{'}}\left(\mathbf{y}-\mathbf{X}\widehat{\boldsymbol{\upbeta}}\right)}{M-P}=\frac{\mathbf{y}\mathbf{\hbox{'}}\mathbf{y}-\widehat{\boldsymbol{\upbeta}}\hbox{'}\mathbf{X}\mathbf{\hbox{'}}\mathbf{y}}{M-P}=\frac{\mathbf{y}\mathbf{\hbox{'}}\mathbf{y}-\mathbf{y}\hbox{'}\mathbf{X}{\left(\mathbf{X}\mathbf{\hbox{'}}\mathbf{X}\right)}^{-\mathbf{1}}\mathbf{X}\mathbf{\hbox{'}}\mathbf{y}}{M-P} $$

The primary focus of this paper is to evaluate the effects of sample size, residual variability, and MSE on the estimated variances $\widehat{\sum}$ of the cell type-specific expression estimates ${\widehat{\beta}}_k$ and the effect this has upon differential expression detection sensitivity.

(Matrix notation for equations 2, 3, 4, 8, 9 and 10 is attributed to Graybill [14]).

### Linear regression-based estimation of differential expression at the cell type-specific level

Differential expression analysis implies comparison of two or more groups for detectable gene expression differences. For simplicity, we consider two-group design, such as a case–control study.

To obtain group specific cell type-specific gene expression estimates $\left({\widehat{\beta}}_{kn}\right)$, we apply linear regression separately to each group of heterogeneous gene expression measures (two regressions). The linear regression coefficient estimates are taken as surrogates for estimated cell type-specific average gene expressions. A difference between these cell type-specific estimates represents the level of gene expression change between the two groups in a given cell type:

11 $$ {\widehat{\delta}}_{kn}={\left(\widehat{\beta}\right)}_{kn}^{cases}-{\left(\widehat{\beta}\right)}_{kn}^{controls} $$

where ${\widehat{\delta}}_{kn}$ is estimated effect size, *k* is the specific cell type and *n* is the genomic site.

Measuring the cell type-specific gene expression differences between groups using linear regression (LR) requires accurate cell type-specific gene expression estimates. Any factors affecting the variability of cell type-specific gene expression estimates per group will affect the sensitivity to detect cell type-specific differences between groups (Fig. 1) (Additional file 1: section 1.2).

### Testing for significant differential expression using two-sample *t*-test

We used Welch’s two-sample *t*-test to determine if the observed effect size (11) is significant (Additional file 1: section 1.3). A per-gene t-statistic is compared against the 1-*α* critical t-value (*α* = 0.05). A t-statistic exceeding the t-critical value is determined to be evidence to reject the null hypothesis of no difference between groups. If a significant difference is determined, sensitivity is then calculated as the upper tail probability beyond the critical *t*-value. If no significant difference is determined, then sensitivity is the alpha level threshold (Fig. 1). LRCDE sensitivity is based upon calculated t-statistic.

### Simulation of cell type-specific expressions with known differential expression

Simulated data was used to assess performance of LRCDE under controlled conditions in which the cell type-specific differential expression was known. To establish a “gold-standard” of known cell type-specific differential expressions to benchmark LRCDE estimates of cell type-specific differential expressions, synthetic data with controlled changes [15] was constructed in three steps.

First, we created synthetic *P* cell proportions with known standard deviation across *M* samples per group for the target cell type *p*. For the sake of comparable per-group regressions, we simulate the condition where both groups have identical cell proportions (Additional file 1: Section 1.4). Second, we created synthetic matrixes of cell type-specific gene expression estimates for both control and case groups. We applied a uniform range of effect sizes (from 0.001 to 1.0) to half of the “genes” in the target cell type *p* of the case group (“true changes”, Additional file 1: Section 1.5). Finally, the cross-product of both synthetic cell type expression and synthetic cell proportion matrices was taken for each group to produce simulated matrices of heterogeneous “fitted values” analogous to the predicted values obtained from linear regression. Normally distributed “noise” was added to the “fitted values” to simulate residual values obtained from a linear regression (Additional file 1: section 1.6). Having these *a priori* known cell type-specific expressions and differential expressions provided us with a benchmark against which to compare the results of LRCDE analysis.

Synthetic data is assembled by joining the two heterogeneous gene expression matrices (“cases” and “controls”) into one 2 *M* by *J* heterogeneous gene expression matrix with a vector of group labels. The two cell proportion matrices, identical for groups of “cases” and “controls” were joined to obtain one 2 *M* by *P* cell proportion matrix.

### Assessing LRCDE sensitivity from simulated data

Simulations over the parameter space were compared using sensitivity based upon simulated (and therefore known) cell type-specific differential expression. To quantify sensitivity, the total number of detected differentially expressed genes was divided by the total number of *a priori* known differentially expressed genes. The sensitivity from simulation using various levels of parameters was compared in order to illustrate the effects of sample size, MSE, and cell proportion variability.

### Parameters affecting cell type-specific expression estimate variance

Parameters that directly impact the variability around cell type-specific expression estimates (linear regression coefficient estimates) include sample size, MSE, and cell type-specific proportion variability across sample (cell proportion SD). For simulations, all but one parameter is held constant. A single simulation is performed for each of a series of discrete values of the parameter of interest. Sensitivity is assessed at each level of the parameter of interest and sensitivity curves are plotted against significance level thresholds for each simulation.

### Assessing the effect of high condition number of cell proportion matrix

A source of variability of cell type specific expression estimates is the “conditioning” or invertibility of the dot-product of the cell proportions predictor matrix. Multivariate linear regression relies upon the dot-product of the predictor matrix, which must be invertible:

12 $$ \mathrm{dot}\ \mathrm{product}=\mathbf{X}\mathbf{\hbox{'}}\mathbf{X} $$

where *X* is the *M* by *P* matrix of cell proportion predictors and *X*’ is the *X* matrix transpose. The inverted matrix is denoted:

13 $$ \mathrm{inverted}\ \mathbf{X}\ \mathrm{matrix}={\left(\mathbf{X}\mathbf{\hbox{'}}\mathbf{X}\right)}^{-1} $$

A non-invertible matrix is referred to as “singular”. A least squares linear regression solution cannot be obtained when the predictor matrix is singular and thus non-invertible. The condition number (CD) of a matrix **X** ' **X** is the ratio of the absolute values of the largest to smallest eigenvalues:

14 $$ CD=\left|\frac{ \max \left( eigen\left(\mathbf{X}\mathbf{\hbox{'}}\mathbf{X}\right)\right)}{ \min \left( eigen\left(\mathbf{X}\mathbf{\hbox{'}}\mathbf{X}\right)\right)}\right| $$

and **X** ' **X** can be factored as:

15 $$ \mathbf{X}\mathbf{\hbox{'}}\mathbf{X}=\mathbf{A}\boldsymbol{\Lambda } \mathbf{A}\mathbf{\hbox{'}} $$

where **Λ** is a diagonal matrix with eigenvalues of **X** ' **X** on the diagonal. Thus, a singular matrix, which has at least one zero eigenvalue, has an undefined condition number. In the case of cell type-specific differential expression detection, the linear regression predictor matrix is the matrix of cell proportions. It is near-singular squared cell proportion predictor matrices which result in unreliable cell expression estimates, and thus unreliable differential expression estimates [16]. Thus, a near-singular cell proportions predictor matrix is a source of cell expression estimate variability [6, 17]. The instability of cell type-specific expression and subsequent differential expression estimates cannot be observed from a single linear regression based upon a single cell proportions predictor matrix. The instability becomes apparent when observing estimates based upon different cell proportions predictor matrices, each with identical standard deviations across samples of the target cell type and identical condition numbers of the squared predictor matrix. It is the exact values comprising the matrices, which vary slightly between matrices (small perturbations) resulting in increasingly greater fluctuations of cell type-specific expression estimates with increasing condition numbers. We aimed at investigating the effect of the condition number for cell proportion on the sensitivity of LRCDE analysis.

Cell proportions were simulated with the target cell standard deviation of 0.2 over samples and a condition number of 100 with 5 total cell types. Group samples sizes were fixed at 10. MSE was fixed at 0.1. Effect size was fixed at 0.2. Allowing the random seed generator to float, this same set of parameters was simulated over 100 iterations. Note that the random seed set prior to the initial iteration in order to allow for replicable results. Thus, a cell proportions predictor matrix with identical target cell standard deviation and condition number of approximately 100 was re-created once for each of the 100 iterations. Letting the random seed float between iterations allowed small perturbations of the cell proportions across all iterations. Each calculated sensitivity observation was collected into a vector and stored for plotting.

Condition numbers of 100, 200, 500, 1000, 5000, 10000, 25000, 50000, and 75000 were simulated and sensitivity was recorded using the same 100-iteration method. The resulting vectors of sensitivity observations for each of the 100 iterations at distinct condition numbers were plotted in adjacent boxplots and variability of sensitivity at each level of condition number was visually compared.

### Dropping cell proportion predictors to address high condition number

As the inherent biological restriction of cell proportions to sum up to 1 leads to high multicollinearity and, consequently, high condition number, reducing multicollinearity may improve the sensitivity of linear regression. We tested the effect of dropping at least one cell proportion predictor with the lowest mean cell in order to reduce multicollinearity. Using target cell proportion standard deviation of 0.1, we tested a model with 5 cell proportions which sum identically to 1 across each sample. These properties of the cell proportion matrix are identical to the model in [5], and result in a condition number of ~75000.

Using simulated conditions, we dropped cell proportions of cell types without introduced differential expression. As dropping a cell type will ultimately affect biological interpretation, we dropped cell types with the lowest mean cell proportions. Intuitively, this may be considered as ignoring potential “noise” in cell proportion measurements.

### Comparing sensitivity of cell type-specific differential expression detection using LRCDE vs. false discovery rate-based methods

False discovery rates (FDR) calculations implemented in the csSAM method [5] rely upon repeated permutation of group membership labels and subsequent repeats of the linear regression cell type-specific differential expression step. A series of 100 cut points is constructed between zero and the greatest cell type-specific differential expression effect size. Differences associated with all genes in a given cell type are then compared against each successive cut point, and the total number of gene differences larger than the cut point is the number of “calls”. The average number of permutation differences across all genes is also compared to this sequence of cut points. For each cut point in each cell type, a potential FDR is calculated by dividing the average number of permutation differences greater than the cut point by the number of calls at the cut point. FDRs are subsequently assigned per gene per cell type by comparing differential expression estimates against cut points and assigning the FDR associated with the greatest cut point smaller than the estimated expression difference.

We contrasted the cell type-specific FDRs with t-statistics *p*-values calculated by the LRCDE approach, which performs analysis on a cell type by cell type gene-by-gene basis. Significance thresholds of 0.0 to 0.3 for FDR and 0.0 to 0.1 (1-*α* for a two-sided test) for t-statistic *p*-value were used to compare true positive rates (TPRs) for both methods tested on the same simulated data over a range differential expression from 0.001 to 1.0. TPRs versus threshold values were then compared graphically for both FDR and t-statistic calculated *p*-values.

### Functional enrichment analysis

Lists of cell type-specific differentially expressed gene names were analyzed using ToppFun module of the ToppGene Suite [18] using default settings.

### Software used for analysis

RStudio [19] v.0.99.491. R packages: GEOquery [20] v.2.36.0, pROC [21] v.1.8, CellMix [22] 1.6.2. CsSAM [5] version 1.2.4, Computer specifications used: Hardware: Intel i7-6700 K 4-core 4.0 GHz, 32 Gb RAM. Operating System: Ubuntu v.15.10, Linux kernel v.4.2.0-35-generic.

## Results

### Parameters affecting sensitivity of linear regression for cell type-specific differential expression detection

Sensitivity (true positive rates – TPR) of linear regression cell type-specific differential expression (LRCDE) detection is affected in two ways. Either, 1) the size of the true differential expression between study groups is changed (effect size), or 2) the variability of cell type-specific expression estimates is changed in one or both study groups.

Variability around cell type-specific expression estimates is affected by three main parameters: sample sizes, residuals sizes (quantified by mean squared error – MSE), and cell type-specific proportion variability across samples. The latter is also dependent on the total number of cell types, five in our study. We found that small changes around this total number of cell types included as predictors had a negligible effect on LRCDE sensitivity (data not shown). Yet, increasing the number of cell types increases the number of predictors in the linear model, making it less parsimonious with respect to the sample size. Furthermore, larger number of cell types decreases cell type-specific proportion variability, and should be avoided. In summary, the variability of cell type-specific expression estimates relative to the size of actual cell type-specific differential expression (eq. 9) drives the sensitivity of differential expression detection (Additional file 1: section 1.2).

### Increased group sample sizes increases sensitivity of LRCDE

Increasing the number of samples in one or both study groups resulted in overall increased sensitivity of differential expression detection, as quantified by TPR curves. As before, other parameters were held fixed. Increasing sample size increases LRCDE sensitivity by reducing variability around cell type-specific expression estimates. Since sample size *M* is in the denominator of the MSE (eq. 10), this result is not surprising. Figure 2a depicts typical increases in TPR observed as sample sizes are increased (Additional file 2: Table S2).

### Increased cell proportion variability across samples increased LRCDE sensitivity

Each cell type used as a predictor in LRCDE always exhibits some degree of variability in its relative proportions across samples. Variability of the proportions of any particular cell type can be quantified by standard deviation. Smaller standard deviation indicates lower variability across samples and conversely larger standard deviation indicates higher variability. In the biologically improbable case of all proportions of a given cell being identical across all samples, then there would be zero variability making linear regression unfeasible.

Cell type-specific differential expression detection sensitivity increases with increased variability of cell type-specific proportions across samples. We simulated cell proportions and tested LRCDE detection sensitivity for several levels of cell proportion variability (Fig. 2b) while holding other parameters fixed (Additional file 3: Table S3). As cell proportion variability across samples is increased, sensitivity of cell type-specific differential expression detection for all genes in that particular cell type improved.

### Reducing mean squared error (MSE) increases sensitivity of LRCDE

Residuals are the differences between actual heterogeneous expression measures (observations) and those same measures as predicted by a regression line (fitted values). Each gene will have a unique set of residuals from linear regression. Sums of squared residuals divided by degrees of freedom (sample size minus the number of cell types) is mean squared error (MSE - numerator of eq. 9). Overall size of residuals as quantified by MSE is one measure of “goodness of fit”, i.e., how well the observed data is predicted by the regression. When other parameters are held fixed (sample sizes, effect size, and cell proportion variability) and MSE is decreased (decreased overall size of residuals), the result is increased differential expression detection sensitivity (Fig. 2c).

Increase in sensitivity is due to the fact that cell type-specific expression estimate variances are decreased proportionately as MSE is decreased (eq. 9). We confirmed this relationship between MSE and variability around cell type-specific expression estimates by simulations (Additional file 4: Table S4). As variability around group-wise cell type-specific expression estimates cover less of a significantly detected difference between these estimates, the sensitivity of differential expression detection increases.

Changes in cell proportion variability for cell type *p* affect the variance (eq. 9) of cell type-specific expression estimates. Since cell proportion variability is captured in the inverse of the design matrix (eq. 9), any increase in cell proportion variability results in a decrease in cell type-specific expression estimate variability. This decrease in cell type-specific expression estimate variability improves sensitivity of LRCDE.

### High condition number of cell proportions predictor matrix results in inconsistent sensitivity

**Fig. 3:**
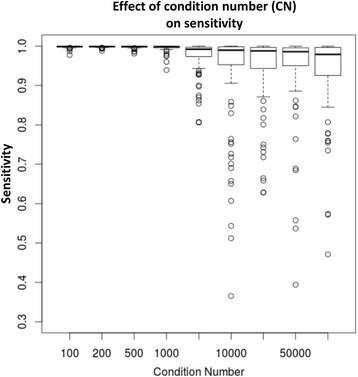
Large condition number of the cell proportion matrix negatively affects stability of sensitivity. Cell proportion matrixes were simulated to obtain condition numbers 100, 200, 500, 1000, 5000, 10000, 25000, 50000, and 75000. Each condition number was simulated 100 times by small perturbations of the cell proportion values. The following parameters were held constant: SD for the target cell proportion – 0.2, per-group sample size – 10, MSE – 0.1, cell type-specific effect size – 0.1

Comparing sensitivity over 100 iterations for each of cell proportions dot product condition numbers of 100, 200, 500, 1000, 5000, 10000, 25000, 50000, and 75000 resulted in fluctuations of t-statistic based sensitivity plotted in Fig. 3. With a condition number of 100, sensitivity remains within a consistent range of values between 0.977 and 0.999. When condition number reaches 1000, we noticed fluctuations of sensitivity from a high of 0.999 to a low of 0.939. At condition number of 5000, the range from maximum sensitivity to minimum had broadened with maximum of 0.999 to minimum of 0.805. When condition number is 10000 maximum sensitivity remains at 0.999 while minimum is 0.364. This loss of consistency of sensitivity with increasing condition number illustrates the instability of an “ill-conditioned” cell proportion matrix.

### Dropping cell proportions with the lowest mean reduces multicollinearity and improves sensitivity

**Fig. 4:**
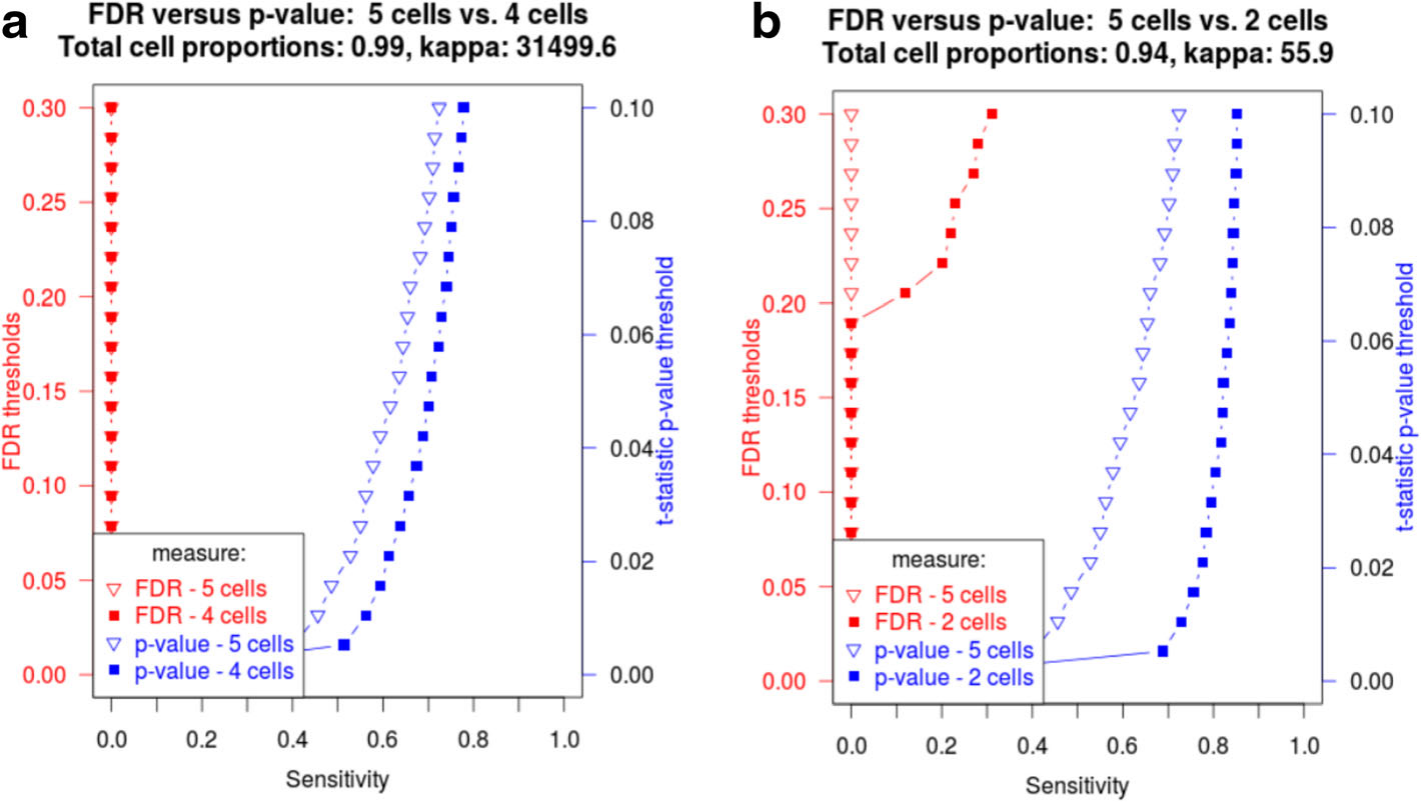
Dropping cell proportions reduces condition number and improves sensitivity. Effect of dropping (**a**) 1 cell type; (**b**) 3 cell proportions with the lowest proportion mean. The following parameters were used: log2-transformed data, per-group sample size – 10, MSE – 1.5, cell type-specific effect ranged from 0.001 to 1.0 over 500 genes out of 1000 simulated genes, SD of the cell type with introduced changes – 0.1, condition number (5 cells total) – 75000

Dropping a single cell type with the lowest proportion mean while retaining ~98 % of total proportions across all samples resulted in reduction of condition number (CD) from ~75000 down to ~31500. This resulted in noticeable improvement in sensitivity of t-statistic *p*-values (Fig. 4a). However, dropping 3 cells while retaining ~94 % of total proportions produced CD of ~56, and, consequently, further improved sensitivity of both FDR and t-statistic *p*-values (Fig. 4b). Although dropping cell types with the lowest proportion mean appears a viable statistical method to improve sensitivity of linear regression-based cell type-specific differential expression analysis, it warrants further investigation of how biological interpretation of the cell proportion estimates is altered.

### Summary of the parameters affecting sensitivity of cell type-specific differential expression

The principal metric driving the sensitivity of LRCDE is the relationship between cell type-specific expression estimate variance and estimated group-wise differential expression. The only way to increase sensitivity of linear regression differential expression detection is to either 1) reduce variances around cell type-specific expression estimates or 2) increase size of cell type-specific differential expression (effect size). While the latter is an experimentally given parameter, the former is affected by several parameters: sample size, sizes of residuals per linear regression as quantified by mean squared error (MSE), or cell type proportion (predictor) variability across samples. These three parameters are the components of variance eq. 9.

### LRCDE accounts for cell type-specific variability of differential gene expression estimate, missed by FDR-based analysis

Figure 2 shows the difference in sensitivity between the FDR-based approach vs. the t-statistic *p*-value approach. Using simulated data, we tested cell type-specific differential expression detection over a range of known effect sizes ranging from 0.001 to 1.0 spread over the 500 changed genes in the target cell. At lower sample sizes, FDR fails to detect any of the known differences below a 0.3 threshold. With sample size of 10 per group (smallest tested), at all but the 0.001 effect size, the t-statistic *p*-value indicates significant changes with sensitivity greater than 0.68 at an alpha significance threshold of 0.025 (for a two-sided test). At 28 samples per group, FDR sensitivity increases from 0 up to 0.848 at a 0.3 threshold. Under the same conditions, t-statistic *p*-value has maximum sensitivity of 0.968, indicating the per-gene significance testing using two-sample *t*-test improves sensitivity of cell type-specific differential expression detection.

All panels in Fig. 2 are representative of the increased sensitivity of the per-gene t-statistics *p*-values vs. FDR. In all cases, the t-statistic *p*-value is more sensitive than FDR to differential expression, particularly in smaller sample sizes. Our results demonstrate that FDR is insensitive to gene-specific variability of cell type-specific expression estimates, leading to higher overall FDRs and thus decreased sensitivity. In contrast, the t-statistic incorporates the variability captured in eq. 2 on a cell-by-cell and gene-by-gene basis.

Ignoring significance thresholds for FDR and t-statistic *p*-values gives the illusion of perfect discrimination or a 100 % true positive rate and 0 % false positive rate (FPR) when analyzing simulated data (Additional file 1: section 1.7). In simulated data, we created the situation in which normally distributed residuals are mean centered around zero. Since linear regression coefficient estimates are unbiased estimates, both regression-based methods will precisely target the known differential expression values, regardless of coefficient variability. For this reason, any known differential expression will always have a lower FDR and a lower t-statistic *p*-value than genes with no differential expression. Thus, in order to truly measure the merits of either method, any measure of differential expression detection performance must be viewed in light of significance thresholds for both FDR and t-statistic *p*-value.

### Biological significance of cell type-specific differentially expressed genes

We compared the performance of per-gene LRCDE analysis with the global FDR threshold-based analysis (csSAM) by analyzing the human whole-blood gene expression measures from 24 kidney transplant patients used by the authors of csSAM [5]. We used liberal FDR < 0.3 threshold for the csSAM method, and the Bonferroni-corrected α = 0.05 level for the *p*-value cutoff in the LRCDE method (Table 1).

**Table 1:** The number of cell type-specific differentially expressed probes (genes) identified in kidney transplant gene expression data from [5]

Cell	Neutrophils	Lymphocytes	Monocytes	Basophils	Eosinophils
Mean	0.592	0.281	0.098	0.025	0.004
SD	0.193	0.151	0.063	0.024	0.003
FDR < 0.3	0	0	1203 (882)	1	0
Bonferroni *p*-value < 0.05	3122	4975	9066 (6018)	648	1263
Overlapping	0	0	1187 (877)	0	0

Mean/SD – overall mean and standard deviation of a cell type-specific proportion

**Fig. 5:**
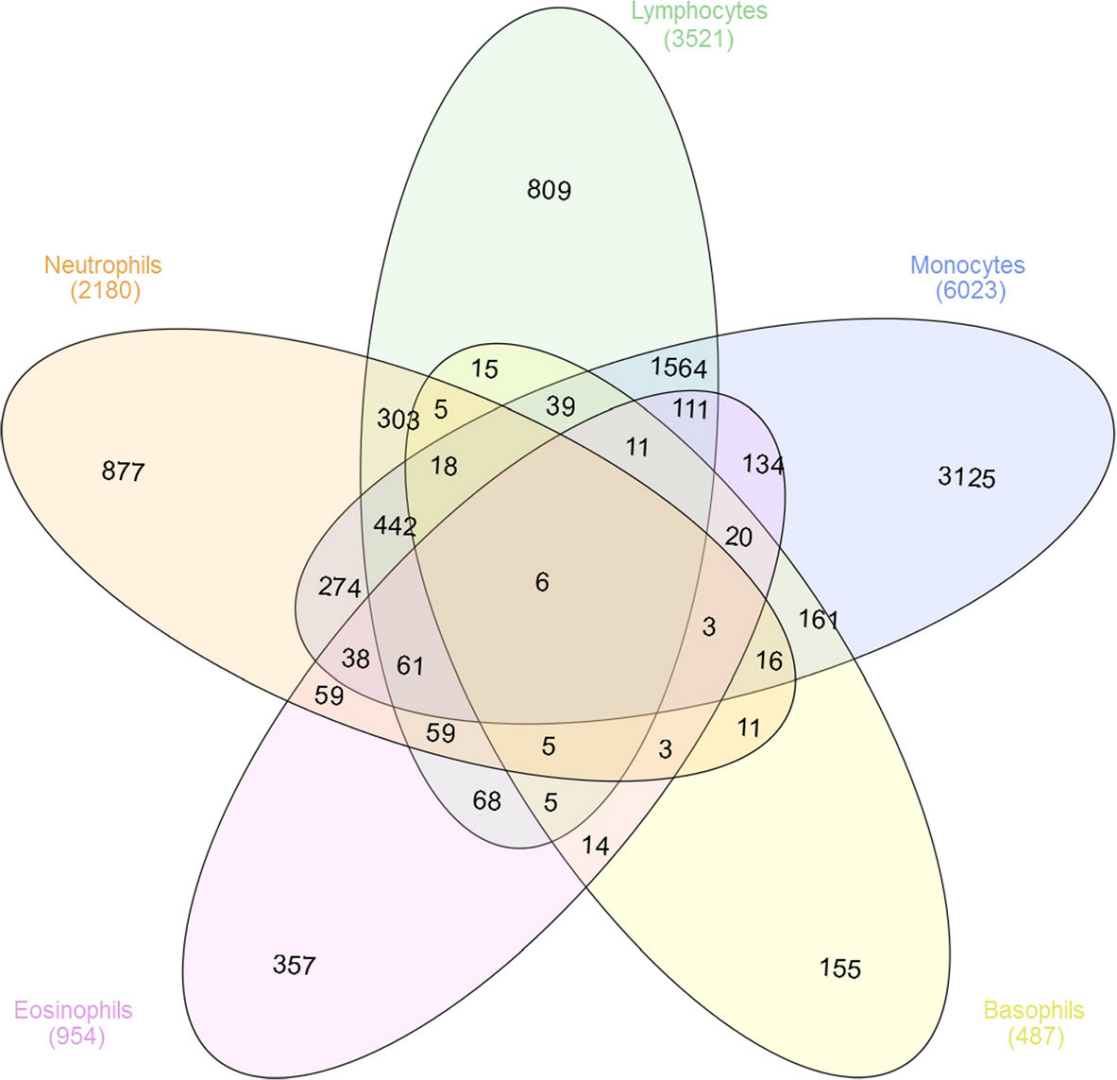
Venn diagram of overlaps among cell type-specific differentially expressed unique gene names in the kidney transplant data set

We identified 59 (10 iterations: 0 to 169) upregulated genes in monocytes at an FDR 0.15, and zero upregulated genes in the other four cell types. Using FDR <0.3, 1203 (10 iterations: 902 to 1696) monocyte-specific genes were detected. In contrast, LRCDE analysis was able to identify significant differentially expressed genes in all five cell types (Table 1, Additional file 5: Table S5, Fig. 5).

Notably, genes detected as differentially expressed in neutrophils were enriched in two functional categories: Ion channel activity/membrane and extracellular matrix/adhesion (Additional file 6: Table S6). Genes detected in lymphocytes and monocytes were enriched in RNA binding/transcription factor activity. Despite being measured in blood, these genes were also enriched in genes involved in kidney regenerative processes (“Human Kidney_Sallustio10_2134genes_DiscriminatedARPCsFromRPTEC/MSC” co-expression category, Additional file 6: Table S6).

Despite many genes were identified as differentially expressed in eosinophils and basophils, they were marginally enriched in processes without obvious biological scheme. This may be attributed to the fact that both cell types had very low mean and SD, making detection of cell type-specific differentially expressed genes less reliable. It remains to be further investigated how the mean/SD of the cell types affect biological outcome of cell type-specific deconvolution.

## Discussion

This paper addresses several key issues of cell type-specific differential expression detection methods based on linear regression (LRCDE). One is the fact that there is a level of uncertainty attached to any detected differential expression at the cell type-specific level, which will change depending upon values of several parameters. Furthermore, this level of uncertainty is different for each gene, and should be accounted for on a per-cell type per-gene basis. The other is the severe multicolinearity of predictors quantified in the condition number of the cell proportions matrix (design matrix).

One of the primary goals of our work has been to quantify the parameter space affecting the sensitivity of LRCDE. One source of variability, the cell proportion measures used as predictor values, affects detection sensitivity within that particular cell type for all genes in the heterogeneous data set. The other source of variability, the mean squared error (MSE - size of residuals) for a given regression, affects the sensitivity for each gene individually.

Characteristics of the cell proportion measures across samples also affect LRCDE detection sensitivity for each gene in the data set. There are two measures of interest. One is the degree of variability for any single cell type across samples. Greater variability of proportion measures across samples will result in higher differential expression detection sensitivity for that specific cell type. Thus, sensitivity will vary from cell type to cell type. This need for variability across samples is acknowledged by the authors of csSAM [5]: “*…accurate estimates of rare cell types may be aided by sample enrichment or inclusion of highly variable samples*”. In this work we have demonstrated the effect of cell type-specific proportion variability upon sensitivity of cell type-specific differential expression detection.

The other measure attached to the cell proportion matrix is a condition number. The condition number of the cell proportion matrix is a global measure of multi-collinearity across all samples and all cell types. It is a function of the ratio of the maximum and minimum eigenvalues of the design matrix. Lower values of the condition number closer to 1 (minimum possible condition number) indicate lower multicollinearity resulting in stable cell type-specific expression estimates (regression coefficients). As the condition number and the variability around cell type-specific estimates are increased, variability of sensitivity also increased. When the condition number associated with the cell proportion predictor matrix is in the tens of thousands then the confidence intervals on the observed sensitivity will increase to the point that conclusive differential expression detection is questionable (Fig. 4). Conditioning of the predictor’s design matrix is therefore a non-trivial source of instability of cell type-specific expression estimates (coefficients estimates from linear regression) and should not be dismissed. Caution is urged when evaluating results of any analysis of linear regression cell type-specific differential expression detection when the cell proportions predictor matrix has a “high condition number” (above 1000). Such “ill-conditioned” cell proportions produce cell type-specific expression estimates which may be unreliable [16], resulting in untrustworthy differential expression estimates. Future use of linear regression techniques for estimation of biological values should take into consideration the condition number of the cell proportion matrix as a source of estimate variability.

Our preliminary investigations of a high multicollinearity and condition number problem suggest that the solution may be simply to drop at least one cell type with the lowest proportion mean. Our results show decreasing condition number and increasing sensitivity of the linear regression-based cell type-specific differential expression analysis. Yet, we have not investigated the biological implications of dropping cell types on cell type-specific expression estimates. We expect that the heterogeneous signal previously allocated to the dropped cell types will then be distributed across the remaining cell type-specific expression estimates. Furthermore, we have only tested the effects of dropping cell types under simulated conditions, when only one cell type contains *a priori* known differentially expressed genes, which is an exception in real biological data. We aim at further investigating the effect of dropping cell types as a means of effectively handling the multicollinearity and large condition number issues without harming biological relevance or cell type-specific differential expression detection.

The practice of log2 transformation of heterogeneous microarray gene measures prior to linear regression deconvolution has been criticized on the basis that log2 transformation of the outcome variable breaks the linear relationship between outcomes and predictors (cell type-specific expression estimates - linear regression coefficients). It has been shown that without applying some back-transformation after performing LRCDE to log2 transformed heterogeneous observations, the cell type-specific expressions will be underestimated [23]. Furthermore, linear regression coefficient estimates may be difficult to interpret in the absence of a linear relationship between the outcome heterogeneous observations and the cell proportion predictors. We tested the effect of log2 transformation on the sensitivity of both FDR-based and LRCDE analyses, but did not identify any measurable sensitivity increase in either method (Fig. 2d). We aim to further investigate the effect of log2 transformation post hoc linear regression by quantifying normality of the residuals and other diagnostic parameters of linear modeling.

The primary drawback of the LRCDE sensitivity calculation is that is relies upon a single linear regression step per group to compute t-statistic based on the standard error estimates of the cell type-specific expressions. This approach carries the implicit assumption of a known distribution of linear regression coefficient estimates. Other algorithms rely upon multiple iterations of the linear regression step in which permutations of group membership labels provides an estimated “null distribution” against which to compare initial estimates. The advantage of these permutation methods is that they do not rely upon assumptions as to the distribution of coefficient estimates. A way to overcome this limitation may be to include facility for a permutation method in which two-sample t-statistics may then be based upon a similar comparison of initial observation versus permuted null observations.

We demonstrated the greater sensitivity to detection of known cell type-specific differential expression using a per-gene two-sample *t*-test approach to differential expression detection. We have drawn attention to parameters affecting gene-specific variability of cell type-specific expression estimates and thus the sensitivity of cell type-specific differential expression detection. Our approach is implemented in an R package, LRCDE, available on GitHub (https://github.com/ERGlass/lrcde.dev), which performs cell type-specific differential expression analysis on a cell type-by-cell type, and gene-by-gene basis. LRCDE estimates cell type-specific differential expression, calculates two-sample t-statistic, t-statistic *p*-value, and, given a significantly detected difference, outputs sensitivity based upon t-statistic (Fig. 1).

## Conclusion

Linear regression-based cell type-specific differential expression (LRCDE) is more sensitive to detect significant cell type specific differential expression than FDR approach. The gene-specific LRCDE sensitivity is a function of sample size, cell type-specific proportion variability, and mean squared error of linear regression for that gene. Larger sample sizes, lower MSE for a linear regression for a given gene, and greater cell type specific proportion variability are needed to achieve greater sensitivity in detecting smaller significant cell type-specific differential expression (smaller effect sizes). The greater the cell proportion variability across samples results in the greater the sensitivity of LRCDE. The magnitude of cell type-specific expression estimate variability relative to size of actual cell type-specific differential expression (eq. 9) drives the sensitivity of differential expression detection. Finally, when the cell proportion matrix has a condition number greater than 1000, then results of LRCDE may be unreliable given the instability introduced by the “ill-conditioned” cell proportions. Preliminary investigation suggests that dropping cell types with low proportion mean increases the sensitivity of linear regression-based cell type-specific differential expression, and should be further investigated.

## Additional files

Additional file 1: Supplementary Methods and Notes. (DOCX 154 kb)
Additional file 2: Table S2. An example of LRCDE and csSAM analysis results of simulated data with 18 samples per group. Target condition number (kappa) – 100, target mean squared error – 1.5, target cell proportion SD – 0.1. Range of the effect sizes introduced in the first 500 genes – 0.001 to 1.0. “site” – Probe ID; “Gene” – Gene label; “base” – estimated non-negative control group cell type-specific expression level; “case” - estimated non-negative case group cell type-specific expression level; “diff.est” – estimated cell type-specific differential expression between case and base; “mse.control” – Observed mean squared error of the control group regression; “mse.case” – Observed mean squared error of the case group regression; “cell” – Cell type name; “cell.sd” – Cell type-specific standard deviation of cell proportions across samples; “kappa.1/2” – Observed condition number of squared cell proportions matrix for the case/control group, respectively; “t.crit” – Critical t-value against which observed t-statistic is compared; “t.stat” – Observed t-statistic; “p.val.t” – Unadjusted *p*-value for t-statistic; “se1/2”– Standard error of the control/case group cell type-specific expression (regression coefficient) estimate, respectively; “se.p” – Welch’s combined standard error calculated for t-statistic; “t.power” – Observed power calculated from t-statistic and critical t-value; “FDR” – False discovery rate as reported by csSAM. (XLSX 136 kb)
Additional file 3: Table S3. An example of LRCDE and csSAM analysis results of simulated data with target cell proportion standard deviation of 0.15. Sample size – 14, target condition number (kappa) – 100, target mean squared error – 1.5. Range of the effect sizes introduced in the first 500 genes – 0.001 to 1.0. Column names legend as in Additional file 1. (XLSX 137 kb)
Additional file 4: Table S4. An example of LRCDE and csSAM analysis results of simulated data with target MSE of 0.5 per regression. Sample size – 14, target condition number (kappa) – 100, target mean squared error – 2.5, target cell proportion SD – 0.06. Range of the effect sizes introduced in the first 500 genes – 0.001 to 1.0. Column names legend as in Additional file 1. (XLSX 138 kb)
Additional file 5: Table 5. LRCDE and csSAM analysis results of the kidney transplant dataset. Column names legend as in Additional file 1. (XLSX 2842 kb)
Additional file 6: Table S6. Functional enrichment analysis of LRCDE significant differentially expressed genes in kidney transplant dataset. “Category” – General functional category; “ID” - unique identifier of the functional category; “Name” – Category name; “Source” – Database reference; “*p*-value” - non-adjusted *p*-value; “q-value FDR B&H” – FDR-adjusted *p*-value; “Hit Count in Query List/Genome” – Number of differential genes/genes in the whole genome annotated with a functional category, respectively; “Hit in Query List” – Names of differentially expressed genes annotated with a functional category. (XLSX 246 kb)

## Abbreviation

CDE: Cell type-specific differential expression
csSAM: Cell-specific significance analysis of microarrays
FC: Fold change
FDR: False discovery rate
FPR: False positive rate
GEO: Gene expression omnibus
LRCDE: Linear regression cell type-specific differential expression
MSE: Mean squared error
ROC: Receiver operator characteristic (curve)
SD: Standard deviation
TPR: True positive rate
