# Supplementary material for: Improving sensitivity of linear regression-based cell type-specific differential expression deconvolution with per-gene vs. global significance threshold
Source: BMC Bioinformatics. 2016 Oct 6;17(Suppl 13):334. doi: 10.1186/s12859-016-1226-z (PMC5073979; doi:10.1186/s12859-016-1226-z)
Supplement: Additional file 1: — Supplementary Methods and Notes. (DOCX 154 kb) [file 12859_2016_1226_MOESM1_ESM.docx]

**Additional file 1**

**1.1 Analysis of Heterogeneous observations versus cell type-specific**

When considering cell type-specific vs. heterogeneous differential expression detection, we may observe two scenarios. In the first scenario, the true difference may exist in only one cell type – it will be detected on heterogeneous level. In the second, the true differences may exist in multiple cell types. If the directionality of those differences is opposite, theoretically they may not be detectable at heterogeneous level by cancelling each other in the cross-product arithmetic. It is for this reason we recommend performing cell type-specific deconvolution on the entire dataset. Selecting the top most varying genes, as done in [5] Supplementary Figure 6, may remove informative signal absent in heterogeneous measures but detectable on cell type-specific level (Table S.1.7).

| **Table S.1.7** Illustration of case in which differential expression is undetectable at heterogeneous level. Given a 2 fold up-regulation of cell 2, then there must be some amount of down-regulation of the same gene in at least one or more other cell types to mask the change at the heterogeneous level. | | | | | | | | | | | | | |
| --- | --- | --- | --- | --- | --- | --- | --- | --- | --- | --- | --- | --- | --- |
|  | Hetero-geneous  Expression gene J |  | Cell 1 Prop |  | cell expression |  | Cell Prop |  | cell expression |  | Cell Prop |  | cell expression |
|  |  |  |  |  |  |  |  |  |  |  |  |  |  |
| controls | **3** | = | **0.2** | x | **2.5** | + | **0.3** | x | **3** | + | **0.4** | x | **4** |
|  | **3** | = | **0.2** | x | **2.5** | + | **0.3** | x | **3** | + | **0.4** | x | **4** |
|  | **3** | = | **0.2** | x | **2.5** | + | **0.3** | x | **3** | + | **0.4** | x | **4** |
|  | **3** | = | **0.2** | x | **2.5** | + | **0.3** | x | **3** | + | **0.4** | x | **4** |
|  | **3** | = | **0.2** | x | **2.5** | + | **0.3** | x | **3** | + | **0.4** | x | **4** |
|  |  |  |  |  |  |  |  |  |  |  |  |  |  |
|  |  |  |  |  |  |  |  |  |  |  |  |  |  |
| cases | **3** | = | **0.2** | x | **0.1** | + | **0.3** | x | **6** | + | **0.4** | x | **2.95** |
|  | **3** | = | **0.2** | x | **0.1** | + | **0.3** | x | **6** | + | **0.4** | x | **2.95** |
|  | **3** | = | **0.2** | x | **0.1** | + | **0.3** | x | **6** | + | **0.4** | x | **2.95** |
|  | **3** | = | **0.2** | x | **0.1** | + | **0.3** | x | **6** | + | **0.4** | x | **2.95** |
|  | **3** | = | **0.2** | x | **0.1** | + | **0.3** | x | **6** | + | **0.4** | x | **2.95** |
|  |  |  |  |  |  |  |  |  |  |  |  |  |  |
|  |  |  | **Fold:** |  | **0.04** |  | **Fold:** |  | **2** |  | **Fold:** |  | **0.7375** |
|  |  |  | **diff:** |  | **-2.4** |  | **diff:** |  | **3** |  | **diff:** |  | **-1.05** |

**1.2 Definition of the parameters affecting cell type-specific expression estimate variability**

The following describes the parameters affecting the estimated variance in case of simple linear regression. The formula for the variance of the linear regression coefficient (single predictor variable *X*) provides an intuitive illustration of how various parameters affect the variance:

$\mathrm{var}\left(\hat{\beta}_{1}\right)=\frac{\sigma^{2}}{\sum_{i=1}^{m} (\mathrm{x}_{i}-\bar{x})^{2}}$ (S1.1).

In practice, the estimated variance of$\hat{\beta}_{1}$ uses the mean squared error (MSE) as an estimate of $\sigma^{2}$, represented as $s^{2}$ :

$s^{2}=MSE=\frac{\sum_{i=1}^{m} (\mathrm{y}_{i}-\hat{\mathrm{y}}_{i})^{2}}{(\mathrm{M}-\mathrm{P})}$ (S1.2).

In this simple linear regression context, *M* is the sample size and *P* is typically equal to 2, since there are two parameters being estimated; an intercept term$\hat{\beta}_{0}$and $\hat{\beta}_{1}$. Thus, the estimated variance of$\hat{\beta}_{1}$ in simple linear regression is represented as:

$\widehat{\mathrm{var}\hat{\beta}_{1}}=\frac{\sum_{i=1}^{m} (\mathrm{y}_{i}-\hat{\mathrm{y}}_{i})^{2}/(\mathrm{M}-\mathrm{P})}{\sum_{i=1}^{m} (\mathrm{x}_{i}-\bar{x})^{2}}$ (S1.3)

where$y_{i}-\hat{y}_{i}$is the residual for sample *i*, and$x_{i}-\bar{x}$ is the difference between the predictor for sample *i* and the mean of *x* across all *M* samples. In this way, predictor variability is captured in the denominator of eq. 7, as is sample size M. Residual variability is captured in the numerator of eq. 7. Each component of eq. 7 affects the estimated variance of $\hat{\beta}_{1}$. These principles are extendable for the case of multivariate linear regression (Methods section).

**1.3 Details of two-sample t-test used in LRCDE**

Any detected differential expression will have variability attached to it as a result of variability around the group-wise cell expression (linear regression coefficient) estimates. In order to test whether an observed difference is statistically significant we apply the Welch-Satterthwaite two-sample t-test, in which we compute the two-sample t-statistic

$t_{diff}=\frac{(\hat{\beta}_{2}-\hat{\beta}_{1})-\delta_{0}}{(\mathrm{se}_{\mathrm{welch}})}$ (S8.1)

for the observed difference and compare it against a t-critical value, where

$se_{welch}=\sqrt{\frac{se_{1}^{2}}{n_{1}}+\frac{se_{2}^{2}}{n_{2}}}$ (S8.2)

and $\delta_{0}=0$ in order to test the null hypothesis of zero difference.

Degrees of freedom used to determine the t-critical value are calculated using Satterthwaite’s equation:

$d.f.=\frac{\left(se_{1}^{2}/n_{1}\right)+\left(se_{2}^{2}/n_{2}\right)}{\left(\frac{\left(se_{1}^{2}/n_{1}\right)^{2}}{(\mathrm{n}_{1}-1)}+\frac{\left(se_{2}^{2}/n_{2}\right)^{2}}{(\mathrm{n}_{2}-1)}\right)}$ (S8.3)

This results in a non-integer degrees of freedom value. Whenever there is a balanced design and both groups have identical standard errors, then the Satterthwaite degrees of freedom will agree with the pooled degrees of freedom:

$d.f._{pooled}=(n_{1}+n_{2}-2)$ (S8.4)

Any difference between group standard errors will result in reduced degrees of freedom in the Satterthwaite equation. In the case that the t-statistic (S8.1) exceeds the t-critical value, then we reject the null hypothesis of no difference and conclude that there is significant evidence indicating that a true difference exists.

In the case of a significant difference, sensitivity is then calculated as the upper tail of the probability distribution based on the distance between the t-statistic (S8.1) and the t-critical value; a negative distance in the case of up-regulation (Figure 1).

**1.4 Simulating cell proportions**

Cell proportions are simulated by creating a per-group *M* by *P* matrix of random uniform values, which must sum to 1 across a row of any given sample [5]. Thus, we have two identical *M* by *P* matrices, one per study group, so that both control group and case group linear regressions are performed with identical cell proportions as predictors.

We chose a target cell standard deviation (SD) to simulate. The target cell is the cell type in the simulation which has a cell type-specific differential expression applied to it in the synthetic cases cell type-specific expression matrix. We then create a single vector of cell proportions for sample size *m*. This begins with a small proportion for sample 1 and creates evenly spaced proportions until a chosen “high” proportion is reached for sample *m*. The SD of this vector is taken and compared to our target SD. If our target SD is too low, we increase the “high” target proportion and recreate the matrix. This iterative brute force process is repeated until we arrive to within a desired tolerance of our target SD.

**1.5 Simulating cell type-specific expression**

1. Choose a constant base level of expression and create a vector of length J of for cell 1
2. Create p-1 remaining cell type expression vectors.
3. Combine cell expression vectors in ‘Cases’ group matrix
4. Copy ‘Cases’ group matrix to create ‘Controls’ group matrix.
5. Alter some percentage (we use 50%) of the “genes” in cell 1 of ‘Cases’ group by applying a fold-change.
6. Make a note of actual known ‘gold-standard’ difference between expression levels of altered genes in cell 1 between ‘Cases’ and ‘Controls’. This is ‘diff’.

**1.6 Simulating heterogeneous observations**

1. Multiply cell proportions matrix by cell type-specific expression matrix: the resulting cross-product equals to the heterogeneous “fitted” values (In real-life these would be the fitted values gotten after performing a linear regression: the “y-hats”).
2. Simulate residuals: either random normal process or empirical residuals.
3. Add simulated residuals to the “fitted” values obtained from step 1.

**1.7 Why zero FPR in FDR and t-statistic differential expression detection**

The Gauss-Markov theorem shows that least squares linear regression provides the best linear unbiased estimator (BLUE) of regression coefficients. LRCDE (t-test) and csSAM (FDR) linear regression coefficient estimates models the average cell type-specific expression levels per group. Increasing or decreasing the variability of coefficient estimates does nothing to alter the unbiasedness of the estimates. Given that the errors have a mean of zero, which we exactly model during simulations, then linear regression will exactly target the true coefficients, which we also exactly model.

Since linear regression coefficient estimates are BLUE, *any* known group-wise differential expression (differences between the two groups coefficient estimates) will result in some noticeable level of detection using FDR threshold or t-statistic. The level of variability of the coefficient estimates will determine the sensitivity of the FDRs or the t-statistics. Given 500 differentially expressed (simulated) genes and 500 unchanged genes in a single cell type, both FDRs and t-statistics will detect a noticeable level of difference in the 500 differentially expressed genes. Neither of them will detect differential expression in the 500 undifferentiated genes.

The csSAM package provides a vector of FDRs for difference estimates for each gene in each cell type. The LRCDE package provides a t-statistic, t-statistic p-value, and sensitivity for each gene in each cell type. Given that each cell type-specific expression (coefficient) estimate is BLUE, then known differential expression will always provide a more significant signal than known lack of differential expression. Using either the FDR vector or a vector from the LRCDE results in order to calculate false positive rates (FPR) will invariably result in a zero FPR for either method.

A hypothetical example will illustrate how both methods produce zero FDRs. It may be that for the 500 known differentiated genes there will be FDRs in the range of 0.9 to 0.95. If we also have FDRs in the range of 0.96 to 0.99 for the known undifferentiated genes, then we have the situation described of having a zero FPR since none of the undifferentiated genes have lower FDRs than the differentiated genes. This applies to t-statistic p-values as well. We may have a vector of t-statistic p-values for the differentiated genes in the range of 0.4 to 0.45 and a range for the undifferentiated genes of 0.46 to 0.49. Such a range of p-values will also produce a zero FPR. Simulation results confirm our reasoning about zero FPRs for both FDRs and t-statistic p-values.

Choosing an acceptable significance threshold for either FDR or LRCDE results will result in less than perfect detection of known differential expression given a range parameter differences: either across a range of known effect sizes, different MSEs, or different cell type-specific standard deviations of cell proportions. Choosing an FDR cutoff threshold of 0.2 and a two-sided t-statistic p-value threshold of 0.025 shows that LRCDE will provide a higher true positive rate (TPR) of discovery than FDR.

The csSAM algorithm computes FDRs by a wholesale comparison of the range of cell type-specific differential expression estimates across all genes in the input data set. FDRs are computed by linearly designated 100 thresholds (cutpoints) across the range of detected effect sizes from lowest to highest. The number of the observed effect sizes greater than each of the cutpoints is stored as a vector of 100 “call” totals. 100 false discovery rates are then computed as the sum of times that the absolute values (for a two-sided test) of permutation based effect sizes are greater than observed (non-permuted) effect sizes divided by the number of permutations divided by the number of calls per cutpoint. Each gene is then assigned one of the 100 FDRs corresponding to the highest of the 100 cutpoints that the effect size is greater than. In this way, FDRs per gene are computed and assigned relative to all genes in the cell.

LRCDE computes a t-statistic, t-statistic p-value, and t-statistic based sensitivity calculation based upon the per gene variability of the group-wise coefficient estimates. The LRCDE approach thus accounts for gene-by-gene differences in the coefficient estimate variability, as well as cell type-specific proportion variability across samples. The more granular approach of LRCDE is thus more sensitive.

**1.8 Comparing analysis of log2 transformed heterogeneous observations versus “as-is” observations in LRCDE analysis**

We compared the TPR of LRCDE analysis of log2 transformed versus “as-is” data, questioned in [23] (Figure 2D). The main argument for using log2 transformed heterogeneous observations in linear regression deconvolution is that log2 transformed observations closely follow a symmetric normal distribution. As the distribution of residuals from linear regression closely follow the distribution of the outcome variable, log2 transformed data satisfies this expectation of linear regression: the normality of residuals.

We performed simulations to address the question of whether analysis of the log2 transformed data provides any advantage over using “as-is” observations. Random normal residuals were applied to the fitted heterogeneous matrix to create heterogeneous observations. These were considered to be the normally distributed log2 transformed heterogeneous observations. These log2 transformed observations were then de-logged to create “as-is” distribution of heterogeneous observations. LRCDE analysis was applied to both log2 transformed and “as-is” heterogeneous matrices using identical simulated cell proportions, and the sensitivity (TPR) of cell type-specific differential expression detection was compared. No substantive difference in TPR was observed between analysis of log2 (normal) versus “delogged” (exponentiated) log2 heterogeneous observations. This is probably a result of the fact that the Gaus-Markov equation demonstrates that linear regression coefficient estimates are the Best Linear Unbiased Estimators (BLUE) of the true unknown coefficients and that there is no strict requirement of a normal distribution of residuals to hold in order for this to be the case.
